# A 48-Year-Old Woman With Shortness of Breath and a Recent Diagnosis of Breast Cancer

**DOI:** 10.1016/j.chpulm.2025.100226

**Published:** 2025-10-27

**Authors:** Sami M. Bennji, Brian Allwood, B. Jayakrishnan, Adil Barakat Al-Riyami, Intisar Yahya, Khalid Al-Baimani, Ahmed Basuoni

**Affiliations:** a Division of Pulmonology, Head & Neck and Thoracic Program, Sultan Qaboos Comprehensive Cancer Care and Research Centre, University Medical City, Muscat, Oman; b Division of Pulmonology, Department of Internal Medicine, Stellenbosch University & Tygerberg Hospital, Cape Town, South Africa; c Division of Cardiology, Internal Medicine, Sultan Qaboos University Hospital, University Medical City, Muscat, Oman; d Breast Program, Sultan Qaboos Comprehensive Cancer Care and Research Centre, University Medical City, Muscat, Oman; e Division of Cardiology, Sultan Qaboos Comprehensive Cancer Care and Research Centre, University Medical City, Muscat, Oman

## Abstract

A 48-year-old perimenopausal woman presented to our hospital in May 2024 with New York Heart Association functional class III dyspnea and a dry cough. Her symptoms had been ongoing for several months but worsened significantly in the weeks before admission. She reported fatigue but denied other constitutional symptoms such as fever, weight loss, or night sweats. There was no orthopnea or paroxysmal nocturnal dyspnea, and she was not taking any medications at the time. She had recently been diagnosed with treatment naïve de novo metastatic left breast cancer, stage cT4bN3cM1, with metastasis to the lungs, liver, bilateral ovaries, and bones. The pathology confirmed invasive ductal carcinoma, grade 1, with estrogen receptor and progesterone receptor positivity and human epidermal growth factor receptor 2-negative status. On presentation, she had not yet received any cancer treatment.

## Physical Examination Findings

On physical examination, the patient appeared mildly distressed. She was tachycardic, with a heart rate of 106 beats/min, and slightly tachypneic, with a respiratory rate of 26 breaths/min. Oxygen saturation was 95% on room air. Cardiovascular examination revealed a raised jugular venous pressure, bilateral lower limb edema, and a loud, split second heart sound (P2).

## Diagnostic Studies

**Figure 1:**
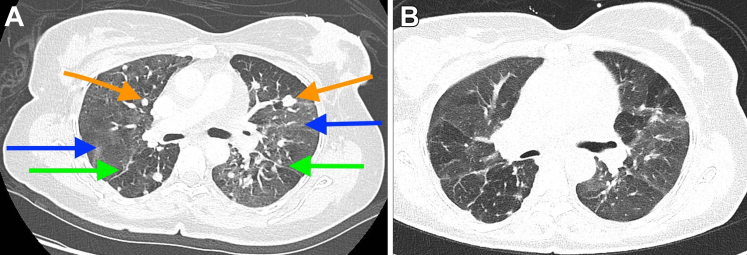
A, CT chest scan showing bilateral ground-glass opacities (blue arrows), pulmonary nodules (orange arrows), and septal thickening (green arrows). B, Follow-up CT scan with resolution of the nodules.

**Figure 2:**
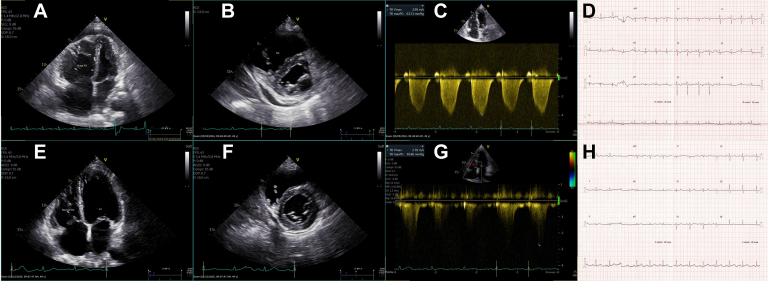
A-D, Echocardiographic and ECG findings on presentation. A, Dilated right ventricle. B, D-shaped septum during systole, indicating pressure overload. C, Continuous Doppler of tricuspid regurgitation jet showing a right ventricular systolic pressure (RVSP) of 64 mm Hg. D, ECG showing right ventricular strain. E-H, Findings after recovery. E, Normal right ventricular size. F, Normal septal shape. G, Marked improvement in RVSP to 32 mm Hg. H, Normalized ECG.

**Figure 3:**
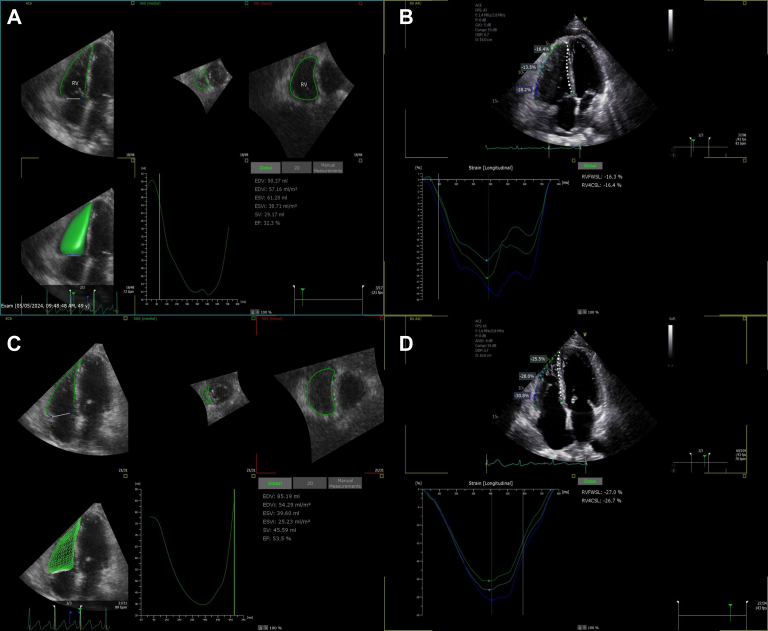
A, B, Right ventricular (RV) function on presentation, with a 4-dimensional (4D) ejection fraction (EF) of 32% and a global longitudinal strain (GLS) of the RV free wall at −16%. C-D, Findings after recovery, with normalization of 4D EF and GLS RV free wall.

Laboratory investigations revealed a pro-B-type natriuretic peptide level of 555 pg/mL (normal < 125 pg/mL). Results of the routine laboratory investigations were unremarkable. CT pulmonary angiography ruled out pulmonary embolism but showed ground-glass opacities, interstitial septal thickening, and pulmonary nodules (Fig 1). ECG showed a right ventricular strain pattern. Transthoracic echocardiography showed a dilated right ventricle with a D-shaped septum during systole, indicating pressure overload, and a peak tricuspid regurgitation velocity of 3.99 m/s (normal value < 2.8 m/s) (Fig 2). Advanced echocardiographic assessment of right ventricular function, including 4-dimensional imaging and global longitudinal strain of the right ventricular free wall, revealed an ejection fraction of 32% (normal > 45%) and a global longitudinal strain of −16% (normal ≤ −21%) (Fig 3).

A comprehensive workup for pulmonary hypertension was initiated. Ventilation-perfusion Single-Photon Emission Computed Tomography–Computed Tomography (SPECT-CT) imaging did not show any perfusion defects and indicated a low probability of pulmonary embolism. Autoimmune and rheumatologic screening, including antinuclear antibody and extractable nuclear antigen profiles, were negative. HIV testing was also negative. The patient was unable to perform pulmonary function tests at that stage.

Right heart catheterization confirmed moderate precapillary pulmonary hypertension with a mean pulmonary artery pressure of 40 mm Hg, pulmonary capillary wedge pressure of 8 mm Hg, cardiac index of 2.5 L/min/m^2^, and pulmonary vascular resistance of 9.1 Wood units (Table 1). A pulmonary artery wedge blood aspiration for cytology was negative for malignant cells.

**Table 1:** Comparative Findings Before and After Recovery From Tumoral Pulmonary Hypertension

Variables	On Presentation	On Recovery
Clinical	Symptoms and signs of right-sided heart failure	Complete recovery of right-sided heart failure
PRO BNP, pg/mL	555	26
Troponin T, ng/L	7	6
ECG	RV strain	Complete recovery
Echocardiography (RV comprehensive assessment)		
RA size	Dilated	Normal
RV size	Dilated dimensions	Normal dimensions
TAPSE, cm	1.1	2.1
Tissue Doppler S wave, cm/s	9	12
PAP, mm Hg	70	40
FAC, %	28	46
RV function by 4D (EF), %	32	56
GLS RV free wall, %	−16	−24
Pericardial effusion	Mild	No effusion
D-shaped septum	Present	Absent PFO is present
Agitated saline	PFO is present	
Right heart catheterization		
Mean PAP, mm Hg	40	20
PCWP, mm Hg	8	8
CI, L/min/m^2^	2.5	2.5
PVR, WU	9.1	3
Qp/Qs	1	1

4D = 4-dimensional; CI = cardiac index; EF = ejection fraction; FAC = fractional area change; GLS = global longitudinal strain; PAP = pulmonary artery pressure; PCWP = pulmonary capillary wedge pressure; PFO = patent foramen ovale; PRO BNP = pro-B-type natriuretic peptide; PVR = pulmonary vascular resistance; Qp/Qs = pulmonary-to-systemic flow ratio; RA = right atrial; RV = right ventricular; TAPSE = tricuspid annular plane systolic excursion; WU = Wood units.

*What is the diagnosis?*

*Diagnosis:* Tumoral pulmonary hypertension, pulmonary tumor thrombotic microangiopathy

## Discussion

Pulmonary hypertension (PH) in patients with cancer can arise from several mechanisms of direct cancer effects due to pulmonary tumor thrombotic microangiopathy, pulmonary tumor emboli, extrinsic compression, or intravascular tumor growth. Additionally, PH may develop as a secondary consequence of cancer therapy. Tumoral PH is a rare manifestation and is often difficult to classify due to various mechanisms involved. Although group I and group IV are often mentioned, it is mostly classified under group V PH. It is mostly associated with metastatic malignancies, notably lung, gastric, and breast cancers. The disease encompasses a spectrum of microvascular involvement ranging from pulmonary tumor thrombotic microangiopathy (PTTM) to pulmonary tumor microembolism. Pathologic features include tumor cell microemboli and microcellular intimal proliferation in small pulmonary veins and arteries. However, the pulmonary vascular remodeling that occurs in PTTM does not appear to involve the formation of plexiform lesions. The condition carries a poor prognosis and is often undiagnosed during life and predominantly confirmed postmortem.

Classic clinical presentation of tumoral PH involves progressive dyspnea, diminished exercise capacity, fatigue, and edema. Although chest CT scan can be normal, characteristic findings like ground-glass opacities, centrilobular nodules, septal thickening, and a tree-in-bud appearance may occasionally occur. Perfusion defects on ventilation-perfusion scan could occur, but CT pulmonary angiography typically does not reveal macroemboli. Although a lung biopsy is required for diagnosis, pulmonary tumor thrombotic microangiopathy could be assumed by methodically ruling out other causes of PH and by establishing the presence of precapillary PH through right heart catheterization. Precapillary PH is defined hemodynamically by a mean pulmonary artery pressure ≥ 20 mm Hg at rest, a pulmonary capillary wedge pressure < 15 mm Hg, and a pulmonary vascular resistance > 2 Wood units. Cytologic findings of malignant cells in wedge aspiration may support the diagnosis, but have a low diagnostic yield ranging from approximately 20% to 30%, based on scattered case reports and small case series.

There is no standardized treatment for PTTM; however, the primary approach involves addressing the underlying malignancy. Case reports have documented recovery after cancer-directed therapy. Adjunctive therapies with tyrosine kinase inhibitors, antivascular endothelial growth factor receptor antibodies, systemic corticosteroids, pulmonary vasodilators, and anticoagulation have also been used.

### Clinical Course

During multidisciplinary team discussion, it was presumed that the PH in the patient was most likely secondary to pulmonary tumor thrombotic microangiopathy. Advanced therapy for PH was not pursued due to the lack of robust evidence supporting its use in this context, and the primary clinical focus remained the management of the newly detected malignancy. Although wedge cytology was negative, the patient demonstrated CT imaging findings consistent with PTTM, and right heart catheterization confirmed precapillary PH. Other potential causes of PH were systematically excluded, supporting the diagnosis. Given her high disease burden, chemotherapy was initiated and she received 6 cycles of docetaxel 75 mg/m^2^ every 3 weeks. Six months later, after completing 6 cycles of chemotherapy, the patient’s symptoms had improved significantly, with dyspnea reduced to New York Heart Association functional class I. Repeat investigations including right heart catheterization showed a marked improvement (Table 1), demonstrating a recovery of tumoral PH after successful cancer treatment. This patient may have presented in the early stages of the remodeling process and a check on the malignant process would have suppressed the ongoing insults or spread of the tumor emboli. Docetaxel could have contributed to the clinical resolution by reducing the tumor burden. Maintenance hormone therapy was commenced with letrozole 2.5 mg daily with ovarian function suppression and ribociclib 600 mg daily for 21 days followed by a 7-day rest; the cycle repeated every 28 days.

## Clinical Pearls

1. *Tumoral PH (group V PH) is a rare and often underrecognized entity. Clinicians should maintain a high index of suspicion in patients with cancer with unexplained dyspnea.*
2. *A systematic diagnostic approach including exclusion of other causes of PH, right heart catheterization, and pulmonary artery blood cytology can aid in diagnosis.*
3. *Prognosis is generally poor; however, significant clinical and hemodynamic improvement is possible with effective cancer treatment.*
4. *The cornerstone of treatment is aggressive treatment of the underlying malignancy.*

## Financial/Nonfinancial Disclosures

None declared.
